# Three-year changes in high-sensitivity cardiac troponin-T and total mortality in older adults

**DOI:** 10.1038/s41598-024-78641-7

**Published:** 2024-11-18

**Authors:** Dhayana Dallmeier, Johanna Braisch, Michael Denkinger, Wolfgang Koenig, Dietrich Rothenbacher

**Affiliations:** 1 Research Unit on Ageing, AGAPLESION Bethesda Clinic Ulm, Ulm, Germany; 2 Medical Faculty, Ulm University, Ulm, Germany; 3 Department of Epidemiology, Boston University School of Public Health, Boston, USA; 4 Institute of Epidemiology and Medical Biometry, Ulm University, Ulm, Germany; 5 Institute for Geriatric Research, Ulm University Medical Center, Ulm, Germany; 6 School of Medicine and Health, German Heart Centre, Technical University of Munich, TUM University Hospital, Munich, Germany; 7 Centre for Cardiovascular Research (DZHK), Partner Site Munich Heart Alliance, Munich, Germany

**Keywords:** Cardiovascular diseases, Prognostic markers

## Abstract

Elevated high-sensitivity cardiac troponin-T (hs-cTnT) is associated with mortality in older adults. However, little is known about the implications of changes over time. We investigated hs-cTnT 3-year change and its association with subsequent mortality in the Activity and Function in the Elderly Study. Participants with baseline and follow-up hs-cTnT < 5 ng/L built the reference group (G1 = 156). Five groups were defined among those with an increment over time: Undetectable at baseline: follow-up < 14 ng/L (G2 = 295), follow-up ≥ 14 ng/L (G3 = 24). Baseline 5 to <14 ng/L: follow-up < 14 ng/L (G4 = 101), follow-up ≥ 14 ng/L (G5 = 96). G6 included baseline and follow-up > 14 ng/L (*n* = 74). Cox-proportional hazards models evaluated the association with mortality adjusting for age, sex, education, cardiovascular disease, chronic kidney disease, number of medications, hs-CRP, and NT-proBNP. Among 745 participants (median age 75.9 years, 58.9% male) we observed 98 deaths (median follow-up 4.8 years). G1 had the lowest mortality rate (MR) (5.2 per 1000 person-years). The highest MR were observed by follow-up ≥ 14 ng/L: G3: 95.4 and G6: 100.4 per 1000 person-years, with an adjusted hazard ratio of 5.22 [95% CI 1.46, 18.65] and 3.40 [95% CI 1.02, 11.34], respectively. Hs-cTnT trajectories could help to identify older adults with a high mortality risk even after further adjustment including hs-CRP and NT-proBNP.

**Supplementary Information:**

The online version contains supplementary material available at 10.1038/s41598-024-78641-7.

## Introduction

Risk stratification represents a crucial part of medical decision making for the identification of patients at highest risk in preventive and clinical medicine. However, tools to identify patients with an increased total mortality risk are scarce and still controversially discussed. Here measurements of additional blood biomarkers may prove useful especially in older adults. Being cardiovascular diseases one of the two leading causes of death among those > 65 years old, understanding the dynamics of cardiac biomarkers as surrogates for cardiovascular disease and total mortality could help to improve such risk stratification.

In this context, cardiac troponin T (cTnT), known to be bound to myofilaments, may play an important role and has already gained widespread recognition for its ability to detect minor myocardial injury. Under normal circumstances, only about 5 to 8% of cTnT is free in the cytosol^1^. The following pathophysiological mechanisms have been discussed for the elevation of hs-cTn: (i) reversible injury to myocyte damage or extracellular vesicle release, (ii) cellular apoptosis or (iii) irreversible cellular damage due to myocardial necrosis^2^. Available high-sensitivity (hs) tests allow the detection of very low concentrations, even in asymptomatic individuals^3,4^. Single elevated levels of hs-cTnT have been identified as important predictors for total mortality even after adjustment for established risk factors and markers for inflammation in the general population, in older adults^4–6^ as well as in non-cardiac populations^7^. Increased cTnT may be related to microvascular coronary disease or unnoticed cardiomyocyte injury observed in conditions such as coronary artery disease, diabetes mellitus, chronic kidney disease, as well as to left-ventricular (LV) strain, decreased subendocardial perfusion, endothelial dysfunction and apoptosis in the setting of heart failure (HF)^6^. In older adults hs-cTnT may be directly associated with changes in LV mass or isolated LV diastolic dysfunction^8^.

Repeated measurements of hs-cTnT have shown an independent association with future cardiovascular events in patients with known cardiovascular disease^9^. Low elevated levels of hs-cTnT may represent myocardial renewal/remodelling or be a sign for subclinical coronary atherosclerosis^10^. Unfortunately, only few studies have examined hs-cTnT trajectories in asymptomatic older adults and therefore data about dynamic changes over time especially in the elderly are rare. We therefore aimed to examine the three-year changes of hs-cTnT in a large population-based cohort of community-dwelling older adults, evaluating the association of identified changes in hs-cTnT levels with subsequent total mortality taking well-established cut-offs for clinical decision beside a continuous analysis into account.

## Methods

### Study populations

The Activity and Function in the Elderly Study (ActiFE) Ulm study is a population-based cohort study in community-dwelling older adults (≥ 65 years) randomly selected in Ulm (Southern Germany) and adjacent regions. Exclusion criteria were severe deficits in cognition, vision, or hearing or serious German language difficulties. ActiFE baseline assessments took place between March 2009 and April 2010 with 1,506 participants (initial response rate was 20% from a population based random sample drawn from the city of Ulm registry office). The study protocol and further details have been published^4,10^. A 3-years follow-up examination was performed between August 2012 and November 2013 with 835 participants. Participants with missing data for identified covariables as well as for hs-cTnT at baseline or follow-up were excluded. In total, 771 participants build the study population for this analysis.

The investigation conforms with the principles outlined in the Declaration of Helsinki. The ethical committees of Ulm University approved the ActiFE study (application no. 318/08 and 50/12). All participants gave written informed consent to participate in the study.

### Data collection

Baseline and follow-up assessments were completed by trained research assistants using standardized methods. The following variables were assessed: age, sex, education, smoking and comorbidities were ascertained by interview-based self-report. The highest completed school education level was binary categorized ($\:\le\:$10 or $\:>$10 years). Smoking was categorized into never, former and current smoker. Comorbidities were assessed by answering the question: “Have you ever been diagnosed with any of the following diseases: hypertension, myocardial infarction, heart failure (HF), stroke, or diabetes”. Information on medications, including name of the medication and number of medications was collected. Body height and weight were measured to calculate body mass index (BMI) in kg/m^2^. BMI was categorized in underweight (< 18.5 kg/m^2^), normal weight ($\:\ge\:\:$18.5 and < 30 kg/m^2^), and obesity ($\:\ge\:\:$30 kg/m^2^). Glomerular filtration rate (GFR) was estimated using the CKD-EPI formula for cystatin C^11^. Systolic and diastolic blood pressures (SBP, DBP) were measured three times and the averages of the last two measurements were used for the analyses.

### Assessment of deaths

Mortality status and date of death were obtained from the local registration offices for all participants with a subsequent median follow-up of 4.8 years.

### Laboratory measurements

At each examination venous blood samples were drawn, immediately centrifuged, processed and stored at -80 °C until analysis^12,13^. All blood samples at baseline and follow-up were drawn between 08:30 and 16:00. No time related change in the hs-cTnT concentrations were detected (Supplementary Fig. 1). hs-cTnT was measured using the Elecsys Troponin T hs Test, with a level of detection (LOD) of 5.0 ng/L and a 10% coefficient of variation (CV) at a level of 13 ng/L. NT-proBNP measurements were performed one year after collection using an electro-chemiluminescence-immunoassay (ECLIA) [Elecsys NT-proBNP II Test, 2010; Roche Diagnostics, Mannheim, Germany; LOD 5 ng/L, measurement range 5-35000 ng/L, intra assay CV 2.4%, inter assay CV 5.6–5.9%]. Differences in the storage time by -80 °C prior to NT-proBNP measurement should not have affected the stability of the biomarker overtime as shown previously^14^. Immunonephelometric assays measured cystatin C [N Latex cystatin C, 2010; Siemens, Eschborn, Germany; LOD 0.05 mg/l, measuring range 0.05–7.25 mg/l, intra assay CV 2.3%, inter assay CV 2.9–3.2%], and serum high-sensitive C-reactive protein (hs-CRP) [N Cardio Phase TM hsCRP, 2010, Behring Nephelometer II, Fa. Siemens, Eschborn, Germany; LOD 0.16 mg/l, measuring range 0.17–1100 mg/l, intra assay CV 3.6%, inter assay CV 5.1–6.7%]. All measures were done batch-wise in a blinded fashion.

### Statistical analyses

Initially descriptive statistics are presented. Continuous variables are described as median with minimum, maximum and interquartile range, or as mean with standard deviation according to their distributions, and categorical variables as numbers with percentages. A total of 475 (63.7%) participants showed hs-cTnT < 5 ng/L at baseline, and 156 (20.9%) at follow-up. These measurements were set at 2.5 ng/L. In addition, the difference between the hs-cTnT at follow-up and baseline as a continuous variable was calculated and the respective descriptive characteristics provided. Due to the skewed distribution of hs-cTnT at baseline and follow-up their values were log-transformed (ln) for the regression analysis.

According to the baseline and follow-up levels of hs-cTnT following six groups (three at baseline and three at follow-up) were build using the LOD of 5 ng/L, an intermediate group with hs-cTnT from 5 to < 14 ng/L, and the third group according to the current ESC Guidelines for the management of acute coronary syndrome with hs-cTnT ≥ 14 ng/L^15^ (details see Table 1). For the 3-years trajectories the reference category was built with those showing baseline and follow-up hs-cTnT < 5 ng/L (undetectable) (Group 1). Five categories were built among those with an increment of hs-cTnT overtime (*n* = 590): For those with undetectable levels at baseline: (i) hs-cTnT at follow-up 5 to < 14 ng/L (Group 2), (ii) hs-cTnT at follow-up ≥ 14 ng/L (Group 3). Among those with baseline levels between 5 to < 14 ng/L: (i) hs-cTnT at follow-up 5 to < 14 ng/L (Group 4), hs-cTnT at follow-up ≥ 14 ng/L (Group 5). Group 6 included those with baseline and follow-up > 14 ng/L.

**Table 1:** Group trajectories according to hs-cTnT levels at baseline and 3-year follow-up.

hs-cTnT at baseline	hs-cTnT at follow-up	Group trajectories
Undetectable (< 5 ng/L)	< 5 ng/L	Group 1
	5 to < 14 ng/L	Group 2
	≥ 14 ng/L	Group 3
5 to < 14 ng/L	5 to < 14 ng/L	Group 4
	≥ 14 ng/L	Group 5
≥ 14 ng/L	≥ 14 ng/L	Group 6

Using Cox proportional hazards models we evaluated the association between the identified groups and total mortality adjusting initially for follow-up information on age and sex (Model 1), followed by additional adjustment for education, history of cardiovascular disease, chronic kidney disease, number of medications (Model 2). We evaluated the association after further adjustment for (ln) hs-CRP and (ln) NT-proBNP (Model 3). In secondary analyses we evaluated the association when using sex-specific cut-offs as suggested by Peacock and colleagues (women 14 ng/l, men 22 ng/l)^16^. In addition, we examined the association between the change in (ln) hs-cTnT as a continuous variable and total mortality for Model 1, Model 2 and Model 3 with additional adjustment for the baseline (ln) hs-cTnT levels after excluding those with undetectable levels at both time points (Group 1) (*n* = 156, 4 deaths). In a sensitivity analysis we evaluated the change in the estimates by excluding those who experienced a relative increment in hs-cTnT < 50% (*n* = 115, 24 deaths), which has been reported in the literature as a clinically not relevant change^3^. By not having information on smoking at follow-up we performed a further secondary analysis adjusting for smoking at baseline under the assumption that smoking behavior in this age was relatively stable through follow-up. Smoking was not identified as an important confounder, as the observed changes in the estimates were all < 10% (see Supplementary Table 2). In a further secondary analysis we evaluated the association of one single measurement of hs-cTnT at follow-up with the subsequent mortality using the established categories (< 5 ng/L, 5 to < 14 ng/L, and ≥ 14 ng/L) for the same models. In addition, we performed a sensitivity analysis by excluding those who at baseline reported a myocardial infarction during the three months prior to the baseline examination (*n* = 2), and/or reported to be hospitalized for a cardiac problem during the month prior to the baseline examination (*n* = 3) or prior to the follow-up examination (*n* = 2) (*n* = 739, 98 deaths for models). The level of significance in the analyses was 5% (two-sided). All analyses were done using SAS version 9.3 (SAS Institute Inc., Cary, North Carolina, USA).

## Results

Table 2 shows participant’s characteristics at 3-years follow-up, which is the starting point for time to death, overall and according to their categories for 3-year trajectory for hs-cTnT. The mean age was 75.9 years (IQR 72.6, 82.2) with 455 (59.0%) male participants. We identified 475 participants (Groups 1–3, 63.7%) with hs-cTnT < 5 ng/L at baseline. From those about one third (*n* = 156) still had hs-cTnT < 5 ng/L at follow-up, who were used as the reference category (Group 1). An increment until < 14 ng/L was observed among 295 participants (Group 2), while 24 participants had hs-cTnT levels ≥ 14 ng/L at follow-up (Group 3). Among those with hs-cTnT at baseline ≥ 5 but < 14 ng/L (*n* = 197), a total of 96 older adults had a further increment of hs-cTnT ≥ 14 ng/L at 3-years follow-up (Group 5). Overall 74 participants had elevated hs-cTnT levels (≥ 14 ng/L) at both time points (Group 6).

The following significant differences were noted between the groups. On average, the youngest participants were observed in the reference group, while the oldest one in Group 6 including those with elevated hs-cTnT levels at both time points. The higher the baseline hs-cTnT levels the higher the proportion of men with only 47.6% among those with hs-cTnT < 5 ng/L, and 83.8% among those with hs-cTnT ≥ 14 ng/L. With respect to comorbidities only those in Group 6 were noted to have a significant increment in the proportion of individuals with cardiovascular diseases (Table 2). As displayed in Supplementary Table 1 all groups showed a significant increment from baseline to follow-up in the proportion of those with eGFR < 60 ml/min per 1.73 m^2^. With the exception of the reference group we observed in all other groups on average an increment in NT-proBNP levels of at least 10% from baseline to follow-up. The NT-proBNP levels were noted to be the highest at baseline and follow-up among those in Group 6. A statistically significant increment in the number of medications could be observed in all 6 groups. However, when evaluating the intake of antihypertensive medication, statins, aspirin or hypoglycemic agents, only a significant increment in the proportion of these medications can be reported for antihypertensive medications.

When evaluated as a continuous variable we observed on average a median change of hs-cTnT of 5.45 ng/L [IQR 3.28, 8.20]. A total of 26 (3.4%) participants were noted to have a decrease of hs-cTnT over time with a median of -1.91 (IQR − 86.73, -3.46). On the other hand, 589 (76.4%) experienced an increment in hs-cTnT levels with a median of 5.67 (IQR 3.50, 8.32), with 156 (20.2%) having no detectable levels to both time points.

**Table 2:** Participant characteristics at 3-years follow-up, starting time point with respect to the subsequent mortality, overall and according to their 3-year hs-cTnT trajectories (*n* = 745).

hs-cTnT at baseline (ng/L)			< 5			[5 to < 14)		≥ 14
hs-cTnT at follow up (ng/L)			$\:<$5	[5 to < 14)	≥ 14	[5 to < 14)	≥ 14	≥ 14
		Study population (*n* = 771)	Group 1 (*n* = 156)	Group 2 (*n* = 295)	Group 3 (*n* = 24)	Group 4 (*n* = 100)	Group 5 (*n* = 96)	Group 6 (*n* = 74)
Number of deaths (%)		101 (13.1)	4 (2.6)	19 (6.4)	9 (37.5)	13 (13.0)	24 (25.0)	29 (39.2)
Mortality rate [95% CI] (per 1000 person-years)		28.5 [23.5, 34.6]	5.2 [2.0, 13.9]	13.5 [8.6, 21.2]	95.4 [49.6, 183.4]	28.2 [16.4, 48.6]	58.9 [39.5, 87.9]	100.4 [69.8, 144.5]
Age, median (q1, q3)		75.9 (72.6, 82.2)	73.5 (71.0, 75.8)	75.0 (72.3, 78.9)	78.5 (74.1, 84.3)	77.5 (73.9, 84.3)	82.4 (76.5, 86.3)	83.0 (76.5, 86.4)
Male, n (%)		455 (59.0)	49 (31.4)	160 (54.2)	17 (70.8)	69 (69.0)	81 (84.4)	62 (83.8)
Smoking at baseline, n (%)	Never	384 (49.9)	85 (54.5)	152 (51.5)	12 (50.0)	46 (46.0)	39 (40.6)	36 (49.3)
	Former	338 (43.9)	61 (39.1)	126 (42.7)	9 (37.5)	46 (46.0)	51 (53.1)	34 (46.6)
	Current	48 (6.2)	10 (6.4)	17 (5.8)	3 (12.5)	8 (8.0)	6 (6.3)	3 (4.1)
Education > 10 years, n (%)		200 (25.9)	41 (26.3)	81 (27.5)	7 (29.2)	29 (29.0)	22 (22.9)	16 (21.6)
BMI kg/m^2^, mean (sd)		27.3 (4.2)	26.4 (4.1)	27.4 (4.0)	28.5 (5.7)	28.0 (4.1)	27.9 (4.0)	27.0 (4.0)
Comorbidities, n (%)								
Cardiovascular disease		245 (31.8)	25 (16.0)	86 (29.2)	12 (50.0)	33 (33.0)	42 (43.8)	38 (51.4)
Myocardial infarction		68 (8.8)	7 (4.5)	19 (6.4)	1 (4.2)	11 (11.0)	15 (15.8)	11 (14.9)
Heart failure		181 (23.5)	18 (11.5)	61 (20.8)	12 (50.0)	22 (22.0)	29 (30.2)	30 (40.5)
Stroke		66 (8.6)	2 (1.3)	24 (8.2)	3 (12.5)	5 (5.0)	11 (11.5)	19 (25.7)
Diabetes		124 (16.1)	14 (9.0)	40 (13.6)	8 (33.3)	21 (21.0)	19 (19.8)	16 (21.6)
GFR ml/min/1.73 m^2^, mean (sd)		70.5 (19.6)	80.8 (16.3)	75.2 (17.5)	57.9 (16.2)	67.6 (16.0)	61.0 (20.9)	52.6 (18.3)
Number of medications, median (q1, q3)		3 (1, 6)	2 (1, 4)	3 (1, 5)	4.5 (3, 7)	4 (2, 6)	4 (2, 6)	6 (4, 8)
hs-CRP, median (q1, q3)		1.3 (0.7, 3.0)	1.1 (0.5, 2.2)	1.2 (0.6, 2.5)	2.1 (1.2, 4.4)	1.9 (0.9, 3.7)	1.9 (0.8, 4.6)	1.6 (0.9, 4.9)
NT-proBNP, median (q1, q3)		160.5 (87.0, 362.3)	105.8 (68.2, 179.6)	135.8 (78.2, 248.3)	231.9 (154.3, 901.1)	165.4 (92.5, 426.7)	338.7 (163.5,600.4)	679.6 (204.6,1606.0)
hs-cTnT at baseline, median (q1, q3)		2.5 (2.5, 8.0)				6.8 (5.9, 8.5)	9.0 (7.0, 11.2)	18.1 (15.3, 24.9)
hs-cTnT at follow-up, median (q1, q3)		8.8 (5.4, 14.5)		7.6 (6.0, 9.2)	16.7 (14.9, 18.5)	11.3 (9.4, 12.7)	17.4 (15.2, 21.7)	30.1 (23.3, 39.6)
Participants with a relative hs-cTnT change ≥ 50%, n (%)		474 (61.5)	0	295 (100.0)	24 (100.0)	46 (46.0)	81 (84.4)	28 (37.8)

**Fig. 1:**
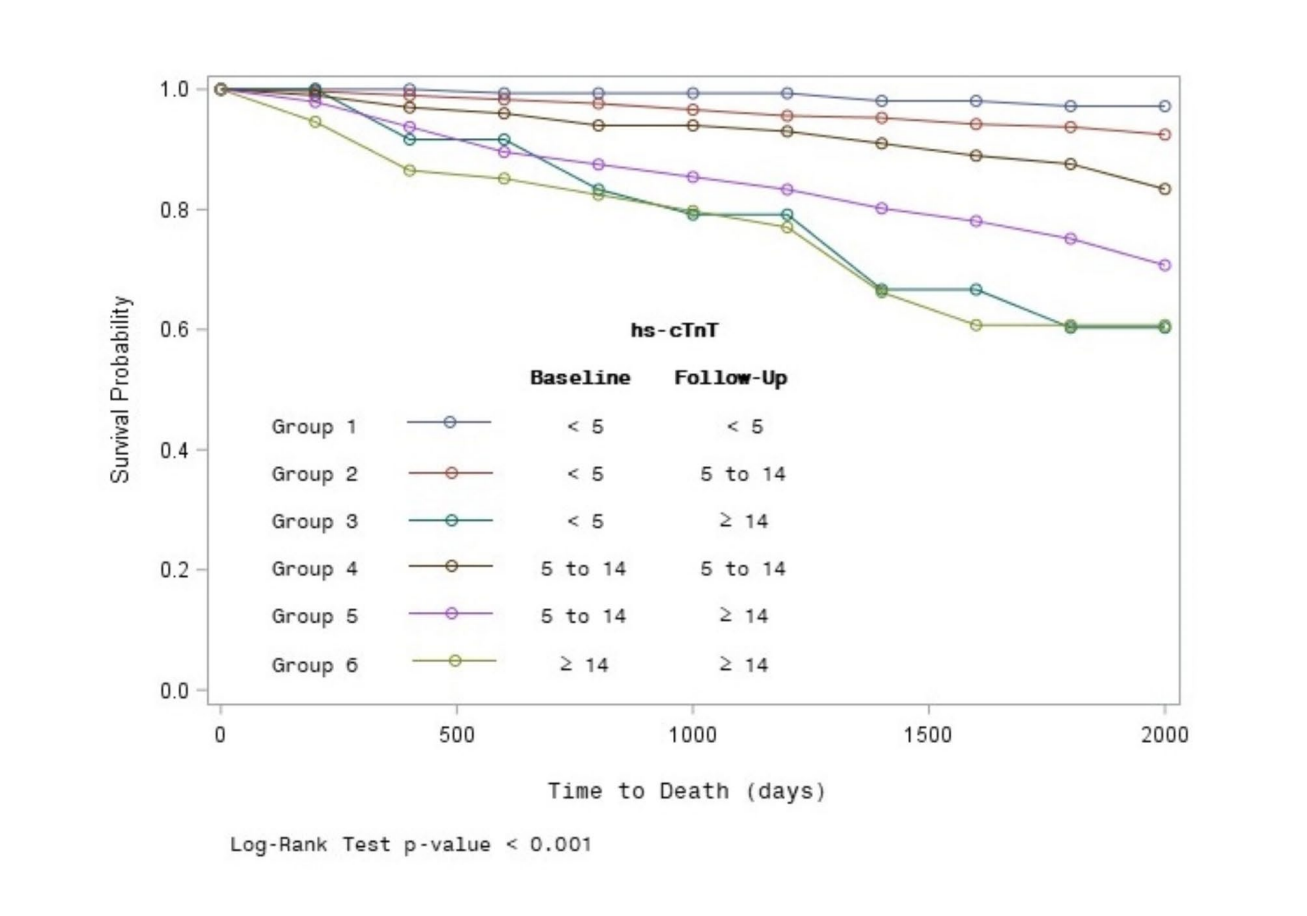
Kaplan–Meier survival curves for each group according to the 3-years hs-cTnT trajectories.

A total of 101 (13.1%) deaths were observed during median follow-up 4.8 years, with an overall mortality rate of 28.5 [95% CI 23.5, 34.6] deaths per 1000 person-years. The lowest mortality rate was found in the reference group with only 5.2 [95% CI 2.0, 13.9] deaths per 1000 person-years. The highest mortality rates were observed among those in Group 6, with baseline hs-cTnT ≥ 14 ng/L and further elevated levels at follow-up, with a mortality rate equal to 100.4 [95% CI 69.8, 144.5] per 1000 person-years, as well as among those with baseline levels < 5 ng/L but follow-up levels ≥ 14 ng/L with a mortality rate of 95.4 [95% CI 49.6, 183.4] per 1000 person-years (Group 3). Figure 1 is showing the Kaplan-Meier survival curves according to the groups (Log-Rank Test p-value < 0.001).

### Multivariable analysis

We detected a significantly increased mortality risk for those in Groups 3, 5 and 6 when compared to the reference groups after adjustment for age and sex with a HR of 8.71[95% CI 2.60, 29.21], 3.33 [95%CI 1.06, 10.51] and 6.00 [95% CI 1.92, 18.75], respectively. The association remained only statistically significant for those in Group 3 and 6 after further adjustment. Adjusted only for clinical covariables we observed a HR of 5.36 [95% CI 1.52, 18.85] and 3.76 [1.15, 12.26] for Groups 3 and 6, respectively. The estimates were attenuated after further adjustment for (ln) hs-CRP and (ln) NT-proBNP remaining statistically significant for Group 3 h 5.22 [95% CI 1.46, 18,65], and Group 6 3.40 [95% CI 1.02, 11.34] (Table 3).

**Table 3:** Multivariable Cox regression evaluating the association between the 3-years hs-cTnT trajectories groups and the subsequent mortality (*n* = 745, 98 deaths), with statistically significant associations in bold.

3-years trajectories Group hs-cTnT values (ng/L) (baseline; follow-up)	Deaths/*n*	HR [95% CI]		
		Model 1	Model 2	Model 3
Group 1 (< 5; < 5)	4/156	Ref	Ref	Ref
Group 2 (< 5; < 14)	19/295	1.61 [0.54, 4.81]	1.39 [0.46, 4.20]	1.39 [0.46, 4.19]
Group 3 (< 5; ≥14)	9/24	**8.71 [2.60**,** 29.21]**	**5.36 [1.52**,** 18.85]**	**5.22 [1.46**,** 18.65]**
Group 4 (5 to < 14; 5 to < 14)	13/101	2.09 [0.64, 6.82]	1.70 [0.51, 5.65]	1.63 [0.49, 5.44]
Group 5 (5 to < 14; ≥14)	24/96	**3.33 [1.06**,** 10.51]**	2.59 [0.81, 8.33]	2.50 [0.77, 8.08]
Group 6 (> 14; ≥14)	29/74	**6.00 [1.92**,** 18.75]**	**3.76 [1.15**,** 12.26]**	**3.40 [1.02**,** 11.34]**

Model 1: adjusted by age and sex.

Model 2: Model 1 plus education, BMI (categ.), cardiovascular disease, diabetes, chronic kidney disease, number of medications (dichotomous).

Model 3: Model 2 plus (ln) hs-CRP und (ln) NT-proBNP.

The sensitivity analysis in a study subpopulation *n* = 630 excluding those with a relative increment of hs-cTnT < 50% showed an increased risk with a HR of 7.06 [95% CI 1.93, 25.86] and 8.55 [95% CI 2.24, 32.66] for those in Groups 3 and 6, respectively, even after adjustment for established clinical risk factors, (ln) hs-CRP and (ln) NT-proBNP (Supplementary Table 3).

### Secondary analyses

The secondary analysis using sex-specific hs-cTnT cut-offs (women 14 ng/L, men 22 ng/L) showed a reduction in the number of those in Group 3, with undetectable levels at baseline but levels higher than the respective cut-offs at follow-up. However, this group can still be identified as the group with the highest risk with a HR of 9.47 [95% CI 2.35, 38.09] even after adjustment for (ln) hs-CRP and (ln) NT-proBNP. An increased risk was also observed among those in Group 5 with a HR of 3.81 [95% CI 1.16, 12.56] (see Supplementary Tables 4 and Supplementary Fig. 2).

The secondary analysis evaluating the association between the change in (ln) hs-cTnT as a continuous variable and total mortality among those with change ≥ 0 showed a statistically significant increased hazard for a one-unit increment in the hs-cTnT levels over time even after adjustment for (ln) hs-CRP and (ln) NTproBNP levels at follow-up (Model 3) with a HR of 1.70 [95% CI 1.04, 2.76] (Supplementary Table 5). Even after inclusion of those with decreasing levels of hs-cTnT overtime for a total *n* = 617 a one-unit increment in the change was associated with an increased hazard with a HR of 1.62 [95% CI 1.92, 2.57] in Model 3 (Supplementary Table 6).

The secondary analysis evaluating the association between hs-cTnT measured just at follow-up and the subsequent mortality showed no significant increased risk in either group with hs-cTnT 5 to < 14 ng/L, and ≥ 14 ng/L after adjustment for covariates when compared to those with undetectable levels (Supplementary Table 7). These results highlight the information gained with respect to mortality risk by considering the three-year trajectory of the biomarker in a study sample of older adults, where competing risk may blunt the effect of the biomarker of interest over time. In addition, the results of the main analysis remained unchanged after excluding those reporting a myocardial infarction at baseline during the three months prior to the baseline examination (*n* = 2), and/or reported to be hospitalized for a cardiac problem during the one month prior to the baseline examination (*n* = 3) or prior to the follow-up examination (*n* = 2) (Supplementary Table 8).

## Discussion

The predictive value of single measurements of hs-cTnT has been reported in several studies including older adults^4,17^. However, the impact of changes over time has been less investigated. To the best of our knowledge this is the first study evaluating longitudinal changes of hs-cTnT in community-dwelling, asymptomatic older adults with respect to total mortality according to the biomarker levels at baseline and 3-years of follow-up. Hereby a small proportion showed undetectable hs-cTnT at both time points. Among those showing an increase of hs-cTnT, those who went from undetectable to hs-cTnT ≥ 14 ng/L showed the strongest increase in mortality even after adjustment for well-established risk factors like hs-CRP and NT-proBNP. Those with hs-cTnT ≥ 14 ng/L at both time point were also identified as a group with a significantly increased risk. Our secondary analysis highlights the added value in the risk stratification by considering longitudinal changes, especially in the presence of competing risk due to the age-related increased mortality. Therefore, considering the trajectories of troponin T may help to identify older adults at higher risk for subsequent mortality, allowing better risk stratification and the implementation of preventive strategies.

The prognostic value of longitudinal measurement of hs-cTnT has been examined across several populations. Data from one study including 176 older patients with stage 4 and 5 of chronic kidney disease showed that longitudinal increases of hs-cTnT were independently associated with all-cause mortality. Other studies performed in dialysis patients have shown this association as well^12,13,18^. When looking at patients with HF an elevation of cTnT over time was also associated with adverse events^3,14,19,20^.

Data from the ARIC Study showed an association between a 6-year hs-cTnT change and the onset of a subsequent events, including coronary heart disease, HF, atrial fibrillation (AF) and death, in asymptomatic middle-aged adults^21,22^. However, the addition of serial measurements of hs-cTnT did not further improve prediction of AF based on the CHARGE-AF score^22^. An association between hs-cTnT changes and subsequent onset of HF and cardiovascular death was also shown in a sample of community-dwelling older adults participating in the Cardiovascular Health Study^3^.

Notably, in our sample participants presenting undetectable levels of hs-cTnT at baseline and levels of hs-cTnT > 14 ng/L showed the highest risk for total mortality even after adjustment for hs-CRP and NT-proBNP. This can suggest that an accelerated progression of myocardial damage may represent a phenotype in older adults with an extreme high risk^21^. Our results reveal the informative gain obtained by performing serial measurements of hs-cTnT even in those asymptomatic older adults, suggesting an improvement in the identification of those with a high mortality risk, a group which could benefit from further diagnostic and therapeutic approaches, that have, however, still to be determined yet.

Statistically significant differences in the distribution of cardiac biomarker in older adults have been reported^4^, pointing out the importance of identifying sex-specific reference values^23^ as well as of evaluating the prognostic value of such biomarkers in men and women respectively. The use of sex-specific cut-offs, as suggested in a study from the US evaluating the predictive value of 3-hr change of hs-cTnT with respect to acute myocardial infarction and major adverse cardiac events^16^, lead in our sample to a reduction in the number of participants with undetectable levels at baseline but levels higher than the respective sex-specific cut-offs at follow-up. Nevertheless, this group could still be identified as the group with the highest mortality risk (Supplementary Tables 4 and Supplementary Fig. 2).

### Strengths and limitations

Our study provides data coming from a moderate sized population-based cohort study of community-dwelling older adults. However, due to its observational nature residual confounding may still be present. Longitudinal measurements of hs-cTnT were performed using the same assay and under similar conditions. Most of participants are Caucasians, therefore our results might not be generalizable to other populations. In addition, no information was collected on smoking at follow-up. However, our secondary analysis using smoking behavior reported at baseline and assuming no change of smoking behavior at follow-up did not show significant changes in estimates, ruling out the need for an additional adjustment for smoking. Unfortunately, we do not have information of any physical activity of moderate or high intensity practiced during the days prior to the follow-up examination, as a possible cause of the observed increment in hs-cTnT as suggested^24^. We were not able to evaluate any cardiovascular events between baseline and 3-year follow-up examinations. Nevertheless, the secondary analysis considering any myocardial infarction or hospitalization due to cardiac reasons in the month prior to each examination show similar results to the main analysis. Unfortunately, we had no cause specific information of death and could therefore only analyze general mortality. In addition, the incidence of cardiovascular diseases such as AF and HF are not available for this cohort. Considering the direct association between high levels of troponin and structural changes in the myocardium one could assume, that the association between longitudinal changes and mortality due to cardiovascular events may even be stronger. Studies testing this hypothesis are needed. Furthermore, the suggested sex-specific cut-offs values were determined in US-patient cohorts with a median age of 55 years and may have limited applicability to our asymptomatic population of older adults.

## Conclusion

Our results showed that a 3-year increment of hs-cTnT is associated with an increased mortality risk even in asymptomatic older adults, suggesting that serial measurements of hs-cTnT may help to identify older adults with a higher mortality risk independent of well-established biomarkers such as hs-CRP and NT-proBNP. These results add value to the available evidence encouraging the performance of clinical studies evaluating the benefit of performing serial measurement of hs-cTnT as a strategy to identify those a highest risk in ambulatory settings followed by the introduction of targeted further diagnostic evaluations as well as interventions adressing changes in lifestyles and optimization of medical treatment.

## Electronic supplementary material

Below is the link to the electronic supplementary material.

Supplementary Material 1

## Data Availability

The datasets generated during and/or analysed during the current study are available from the corresponding author on reasonable request.
