# Supplementary material for: Three-year changes in high-sensitivity cardiac troponin-T and total mortality in older adults
Source: Sci Rep. 2024 Nov 18;14:28412. doi: 10.1038/s41598-024-78641-7 (PMC11574211; doi:10.1038/s41598-024-78641-7)

Three-year changes in high-sensitivity cardiac troponin-T and total mortality in older adults.

Dhayana Dallmeier*[1,2,3], Johanna Braisch[1,4], Michael Denkinger[1,5], Wolfgang Koenig[4,6,7], Dietrich Rothenbacher[4].

Affiliations:
1 Research Unit on Ageing, AGAPLESION Bethesda Clinic, Ulm, Germany
2 Medical Faculty, Ulm University, Ulm, Germany
3 Department of Epidemiology, Boston University School of Public Health, Boston, USA
4 Institute of Epidemiology and Medical Biometry, Ulm University, Ulm, Germany
5 Institute for Geriatric Research, Ulm University Medical Center, Ulm, Germany
6 School of Medicine and Health, Technical University of Munich, TUM University Hospital, Munich, Germany;
7 Centre for Cardiovascular Research (DZHK), partner site Munich Heart Alliance, Munich Germany

Supplementary Material

Author for correspondence
Dhayana Dallmeier, MD PhD
Research Unit on Ageing
AGAPLESION Bethesda Clinic, Ulm
Zollernring 26
89073 Ulm, Germany
Tel: +49731187190
ddallmei@bu.edu

Supplementary Table 1. Participant characteristics at baseline and three-years follow-up according to their hs-cTnT Trajectories (n=746)

			**Baseline Levels < 5 ng/L**			**Baseline levels 5 ng/L to 14 ng/L**		**Baseline Levels ≥ 14**
		**Exam***	**Group 1 (n=156):** Follow-Up Levels < 5 ng/L	**Group 2 (n=295):** Follow-Up Levels 5 ng/L to 14 ng/L	**Group 3 (n=24):** Follow-Up Levels ≥ 14 ng/L	**Group 4 (n=100):** Follow-Up Levels 5 ng/L to 14 ng/L	**Group 5 (n=96):** Follow-Up Levels ≥ 14 ng/L	**Group 6 (n=74):** Follow-Up Levels ≥ 14 ng/L
Age, median (min, q1, q3, max)		BL	70.2 (65.4, 67.7, 72.5, 86.4)	71.7 (65.4, 69.0, 75.6, 89.7)	74.9 (67.5, 70.7, 80.9, 87.0)	74.2 (65.4, 70.6, 81.9, 90.3)	79.1 (66.9, 73.2, 83.0, 91.5)	79.7 (66.3, 73.2, 83.1, 90.3)
		FU	73.5 (69.0, 71.0, 75.8, 89.4)	75.0 (68.7, 72.3, 78.9, 93.3)	78.5 (70.8, 74.1, 84.3, 90.6)	77.5 (69.0, 73.9, 84.3, 93.6)	82.4 (70.2, 76.5, 86.3, 94.8)	83.0 (69.6, 76.5, 86.4, 93.6)
Male, n (%)		BL	49 (31.4)	160 (54.2)	17 (70.8)	69 (69.0)	81 (84.4)	62 (83.8)
Smoking, n (%)	never	BL	85 (54.5)	152 (51.5)	12 (50.0)	46 (46.0)	39 (40.6)	36 (49.3)
	former		61 (39.1)	126 (42.7)	9 (37.5)	46 (46.0)	51 (53.1)	34 (46.6)
	current		10 (6.4)	17 (5.8)	3 (12.5)	8 (8.0)	6 (6.3)	3 (4.1)
Education > 10 years, n (%)		BL	41 (26.3)	81 (27.5)	7 (29.2)	29 (29.0)	22 (22.9)	16 (21.6)
BMI kg/m^2, mean (sd)		BL	26.5 (4.2)	27.3 (3.8)	28.6 (5.3)	28.2 (4.0)	28.35 (3.75)	27.73 (3.76)
		FU	26.4 (4.1)	27.4 (4.0)	28.5 (5.7)	28.0 (4.1)	27.89 (4.04)	27.02 (4.05)
Cardiovascular disease, n (%)		BL	18 (11.5)	66 (22.4)	8 (33.3)	22 (22.0)	33 (34.4)	25 (33.8)
		FU	25 (16.0)	86 (29.2)	12 (50.0)	33 (33.0)	42 (43.8)	38 (51.4)
Myocardial infarction		BL	6 (3.9)	16 (5.4)	1 (4.2)	10 (10.0)	13 (13.5)	7 (9.5)
		FU	7 (4.5)	19 (6.4)	1 (4.2)	11 (11.0)	15 (15.8)	11 (14.9)
Heart failure		BL	13 (8.3)	41 (13.9)	7 (29.2)	11 (11.0)	20 (20.8)	13 (17.6)
		FU	18 (11.5)	61 (20.8)	12 (50.0)	22 (22.0)	29 (30.2)	30 (40.5)

Stroke	BL	1 (0.6)	14 (4.8)	2 (8.3)	3 (3.0)	5 (5.2)	12 (16.2)
	FU	2 (1.3)	24 (8.2)	3 (12.5)	5 (5.0)	11 (11.5)	19 (25.7)
Diabetes, n (%)	BL	11 (7.1)	28 (9.5)	5 (20.8)	16 (16.0)	16 (16.7)	13 (17.6)
	FU	14 (9.0)	40 (13.6)	8 (33.3)	21 (21.0)	19 (19.8)	16 (21.6)
Chronic kidney disease (GFR<60 ml/min/1,73 m^2), n (%)	BL	3 (1.9)	15 (5.1)	4 (16.7)	11 (11.0)	20 (20.8)	20 (27.0)
	FU	19 (12.2)	60 (20.3)	13 (54.2)	33 (33.0)	41 (42.7)	46 (62.2)
Number of medications, median (min, q1, q3, max)	BL	2 (0, 1, 3, 10)	2 (0, 1, 4, 13)	4 (0, 2, 6, 8)	3 (0, 1.5, 5, 10)	3.5 (0, 1, 6, 11)	5 (0, 2, 7, 14)
	FU	2 (0, 1, 4, 12)	3 (0, 1, 5, 12)	4.5 (0, 3, 7, 13)	4 (0, 2, 6, 10)	4 (0, 2, 6, 13)	6 (0, 4, 8, 13)
Anti-hypertensive medication, n (%)	BL	62 (39.7)	164 (55.6)	17 (70.8)	67 (67.0)	63 (65.6)	55 (74.3)
	FU	74 (47.4)	195 (66.1)	17 (70.8)	72 (72.0)	68 (70.8)	59 (79.7)
Statine, n (%)	BL	32 (20.5)	78 (26.4)	6 (25.0)	24 (24.0)	29 (30.2)	23 (31.1)
	FU	35 (22.4)	88 (29.8)	8 (33.3)	33 (33.0)	33 (34.4)	24 (32.4)
Aspirin, n (%)	BL	18 (11.5)	74 (25.1)	9 (37.5)	35 (35.0)	20 (20.8)	34 (46.0)
	FU	27 (17.3)	83 (28.1)	5 (20.8)	34 (34.0)	33 (34.4)	30 (40.5)
Diabetes medication, n (%)	BL	3 (1.9)	16 (5.4)	4 (16.7)	13 (13.0)	13 (13.5)	9 (12.2)
	FU	5 (3.2)	20 (6.8)	7 (29.2)	14 (14.0)	12 (12.5)	12 (16.2)
hs-CRP, median (min, q1, q3, max)	BL	1.2 (0.1, 0.6, 2.4, 39.6)	1.5 (0.1, 0.8, 3.3, 34.8)	2.2 (0.5, 1.1, 3.4, 27.7)	2.0 (0.1, 1.2, 4.3, 27.5)	1.7 (0.1, 0.9, 3.4, 60.9)	1.8 (0.2, 1.0, 3.6, 26.0)
	FU	1.1 (0.1, 0.5, 2.2, 17.8)	1.2 (0.1, 0.6, 2.5, 42.7)	2.1 (0.4, 1.2, 4.4, 7.2)	1.8 (0.1, 0.9, 3.7, 46.5)	1.9 (0.1, 0.8, 4.6, 62.0)	1.6 (0.3, 0.9, 4.9, 50.1)
NT-proBNP, median (min, q1, q3, max)	BL	103.5 (2.5, 56.9, 170.0, 995.0)	109.0 (5.6, 63.9, 192.0, 2293.0)	163.5 (13.4, 93.6, 235.0, 1824.0)	150.5 (9.1, 75.1, 283.0, 1742.0)	209.0	438.0

						(2.5, 108, 326.0, 3042.0)	(23.8, 194.0, 814.0, 2215.0)
	FU	105.8 (7.1, 68.2, 179.6, 1378.0)	135.8 (12.2, 78.2, 248.3, 2367.0)	231.9 (27.9, 154.3, 901.1, 1884.0)	165.4 (17.6, 92.5, 426.7, 3739.0)	338.7 (37.2, 163.5 ,600.4, 8626.0)	679.6 (45.0, 204.6, 1606.0, 15249.0)
hs-Troponin T, median (min, q1, q3, max)	BL				6.9 (5.0, 5.9, 8.5, 12.4)	9.0 (5.0, 7.0, 11.2, 13.9)	18.1 (14.1, 15.3, 24.9, 47.2)
	FU		7.6 (5.0, 6.0, 9.2, 14.0)	16.7 (14.0, 14.9, 18.5, 35.8)	11.2 (5.7, 9.5, 12.7, 14.0)	17.4 (14.0, 15.2, 21.7, 48.2)	30.1 (15.2, 23.3, 39.6, 393.6)
Number of subjects with a relative hs-cTnT change ≥ 50%, n(%)		0	295 (100.0)	24 (100.0)	46 (46.0)	81 (84.4)	28 (37.8)
Number of deaths (%)		4 (2.6)	19 (6.4)	9 (37.5)	13 (13.0)	24 (25.0)	29 (39.2)
Mortality rate [95% CI] (per 1000 person-years)		5.2 [2.0, 13.9]	13.5 [8.6, 21.2]	95.4 [49.6, 183.4]	28.2 [16.4, 48.6]	58.9 [39.5, 87.9]	100.4 [69.8, 144.5]

* Exam: BL= Baseline Examination, FU = Follow-up Examination

Supplementary Table 2: Sensitivity analyses with **smoking at baseline** (never/former/current smoker).

**3-years trajectories Group hs-CTnT values (ng/L) (baseline; follow-up)**	**HR [95% CI]**			
	**Model 2**		**Model 3**	
	**without Smoking**	**with smoking**	**without Smoking**	**with smoking**
**Group 1 (<5; < 5)**	Ref	Ref	Ref	
**Group 2 (<5; < 14)**	1.39 [0.46, 4.20]	1.47 [0.49, 4.46]	1.39 [0.46, 4.19]	1.42 [0.47, 4.28]
**Group 3 (< 5; ≥14)**	**5.36 [1.52, 18.85]**	**6.32 [1.81, 22.10]**	**5.22 [1.46, 18.65]**	**5.26 [1.47, 18.79]**
**Group 4 (5 to <14; 5 to <14)**	1.70 [0.51, 5.65]	2.01 [0.61, 6.62]	1.63 [0.49, 5.44]	1.68 [0.50, 5.61]
**Group 5 (5 to <14; ≥14)**	2.59 [0.81, 8.33]	3.19 [1.00, 10.20]	2.50 [0.77, 8.08]	2.56 [0.79, 8.29]
**Group 6 (>14; ≥14)**	**3.76 [1.15, 12.26]**	**5.35 [1.65, 17.41]**	**3.40 [1.02, 11.34]**	**3.56 [1.06, 11.98]**
**deaths/n**	98/745	97/744	98/745	97/744

Model 2: Model 1 plus education, BMI (categ.), cardiovascular disease, diabetes, chronic kidney disease, number of medications (dichotom)
Model 3: Model 2 plus (ln) hs-CRP und (ln) NT-proBNP

Supplementary Table 3. Secondary analysis among those with an increment of at least 50% (n=630, 74 deaths).

**3-years trajectories Group hs-CTnT values (ng/L) (baseline; follow-up)**	**deaths/n**	**HR [95% CI]**		
		**Model 1**	**Model 2**	**Model 3**
**Group 1 (<5; < 5)**	4/156	Ref	Ref	Ref
**Group 2 (<5; < 14)**	19/295	1.59 [0.53, 4.78]	1.48[0.49, 4.51]	1.42 [0.47, 4.33]
**Group 3 (< 5; ≥14)**	9/24	**8.80 [2.60, 29.81]**	**5.88 [1.62, 21.33]**	**7.06 [1.93, 25.86]**
**Group 4 (5 to <14; 5 to <14)**	5/46	1.45 [0.36, 5.83]	1.24 [0.30, 5.06]	1.25 [0.30, 5.17]
**Group 5 (5 to <14; ≥14)**	22/81	**3.49 [1.06, 11.48]**	3.04 [0.91, 10.20]	3.25 [0.97, 10.85]
**Group 6 (>14; ≥14)**	15/28	**9.24 [2.73, 31.21]**	**6.82 [1.86, 25.06]**	**8.55 [2.24, 32.66]**

Model 1: adjusted by age and sex
Model 2: Model 1 plus education, BMI (categ.), cardiovascular disease, diabetes, chronic kidney disease, number of medications (dichotom)
Model 3: Model 2 plus (ln) hs-CRP und (ln) NT-proBNP

Supplementary Table 4. Secondary analysis using sex-specific cut-offs (n=745, 98 deaths).

3-years trajectories Group hs-CTnT values (ng/L) (death/n)	Sex	Sex-specific cut-offs	Sex-specific deaths/n	HR [95% CI]		
				Model 1	Model 2	Model 3
**Group 1** (4/156)	male	(<5; < 5)	2/49	Ref	Ref	Ref
	women	(<5; < 5)	2/107			
**Group 2** (23/310)	male	(<5; < 22)	12/175	1.71 [0.58, 5.04]	1.46 [0.49, 4.34]	1.42 [0.47, 4.22]
	women	(<5; < 14)	11/135			
**Group 3** (5/9)	male	(< 5; ≥22)	2/2	**11.69 [3.02, 45.35]**	**9.31 [2.32, 37.38]**	**9.47 [2.35, 38.09]**
	women	(< 5; ≥14)	3/7			
**Group 4** (27/177)	male	(5 to <22; 5 to <22)	25/146	1.82 [0.59, 5.65]	1.50 [0.48, 4.71]	1.43 [0.45, 4.52]
	women	(5 to <14; 5 to <14)	2/31			
**Group 5** (25/57)	male	(5 to <22; ≥22)	21/42	**6.02 [1.95, 18.63]**	**4.17 [1.29, 13.50]**	**3.81 [1.16, 12.56]**
	women	(5 to <14; ≥14)	4/15			
**Group 6** (14/36)	male	(>22; ≥22)	9/24	**4.90 [1.49, 16.13]**	3.17 [0.90, 11.10]	2.46 [0.67, 9.00]
	women	(>14; ≥14)	5/12			

Model 1: adjusted by age and sex
Model 2: Model 1 plus education, BMI (categ.), cardiovascular disease, diabetes, chronic kidney disease, number of medications (dichotom)
Model 3: Model 2 plus (ln) hs-CRP und (ln) NT-proBNP

**Supplementary Table 5.** Secondary analysis evaluating the association between the change in log-hs-cTnT as a continuous variable and total mortality among those with change ≥ 0 (n=589, 94 deaths).			
**HR [95% CI]**	**Model 1**	**Model 2**	**Model 3**
**log-transformed hs-cTnT per unit change**	**1.93 [1.32, 2.81]**	**1.79 [1.13, 2.83]**	**1.70 [1.04, 2.76]**
**Model 1:** adjusted by ln-hs-cTnT at baseline, age and sex<br>**Model 2:** Model 1 plus education, BMI (categ.), cardiovascular disease, diabetes, chronic kidney disease, number of medications (dichotom)<br>**Model 3:** Model 2 plus (ln) hs-CRP und (ln) NT-proBNP			

**Supplementary Table 6.** Secondary analysis evaluating the association between the change in log-hs-cTnT as a continuous variable and total mortality after exclusion of only those with undetectable levels of the biomarkers at both time point (n=617, 97 deaths)			
**HR [95% CI]**	**Model 1**	**Model 2**	**Model 3**
**log-transformed hs-cTnT change**	**1.96 [1.38, 2.78]**	**1.72 [1.11, 2.65]**	**1.62 [1.02, 2.57]**
**Model 1:** adjusted by ln-hs-cTnT at baseline, age and sex<br>**Model 2:** Model 1 plus education, BMI (categ.), cardiovascular disease, diabetes, chronic kidney disease, number of medications (dichotom)<br>**Model 3:** Model 2 plus (ln) hs-CRP und (ln) NT-proBNP			

Supplementary Table 7: Multivariable Cox Regression evaluating the association between hs-cTnT at three years follow-up and the subsequent mortality (n=771, 101 deaths).

Group hs-cTnT follow-up values (ng/L)	deaths/n	HR [95% CI]		
		Model 1	Model 2	Model 3
**<5**	4/159	Ref	Ref	Ref
**5 to < 14**	33/405	1.77 [0.61, 5.14]	1.46 [0.50, 4.27]	1.44 [0.49, 4.22]
**≥14**	64/207	**4.61 [1.55, 13.65]**	3.04 [0.998, 9.24]	2.85 [0.93, 8.77]

Model 1: adjusted for age and sex
Model 2: Model 1 plus education, BMI (categ.), cardiovascular disease, diabetes, chronic kidney disease, number of medications (dichotom)
Model 3: Model 2 plus (ln) hs-CRP und (ln) NT-proBNP

Supplementary Table 8: Secondary Analysis excluding those reporting to have a myocardial infarction during the prior three months (n=2), and/or reported to be hospitalized for a cardiac problem during the one month prior to baseline examination (n=3) or reported to be hospitalized for a cardiac problem during the one month prior to follow-up examination (n=2) (n=739, 98 deaths)

**3-years trajectories Group hs-cTnT values (ng/L) (baseline; follow-up)**	**deaths/n**	**HR [95% CI]**		
		**Model 1**	**Model 2**	**Model 3**
**Group 1 (<5; < 5)**	4/156	Ref	Ref	Ref
**Group 2 (<5; < 14)**	19/292	1.63 [0.55, 4.89]	1.46 [0.49, 4.38]	1.45 [0.49, 4.35]
**Group 3 (< 5; ≥14)**	9/23	**9.05 [2.69, 30.42]**	**6.00 [1.76, 20.46]**	**5.80 [1.69, 19.89]**
**Group 4 (5 to <14; 5 to <14)**	13/98	2.14 [0.65, 6.98]	1.90 [0.60, 6.04]	1.80 [0.57, 5.74]
**Group 5 (5 to <14; ≥14)**	24/96	**3.35 [1.06, 10.57]**	2.86 [0.94, 8.73]	2.73 [0.89, 8.32]
**Group 6 (>14; ≥14)**	29/74	**6.03 [1.93, 18.84]**	**4.22 [1.38, 12.89]**	**3.76 [1.21, 11.66]**

Model 1: adjusted by age and sex
Model 2: Model 1 plus education, BMI (categ.), cardiovascular disease, diabetes, chronic kidney disease, number of medications (dichotomous)
Model 3: Model 2 plus (ln) hs-CRP und (ln) NT-proBNP

Supplementary Figure 1. Log-transformed hs-cTnT versus time of blood draw a) at baseline, b) at 3-years follow-up

a)

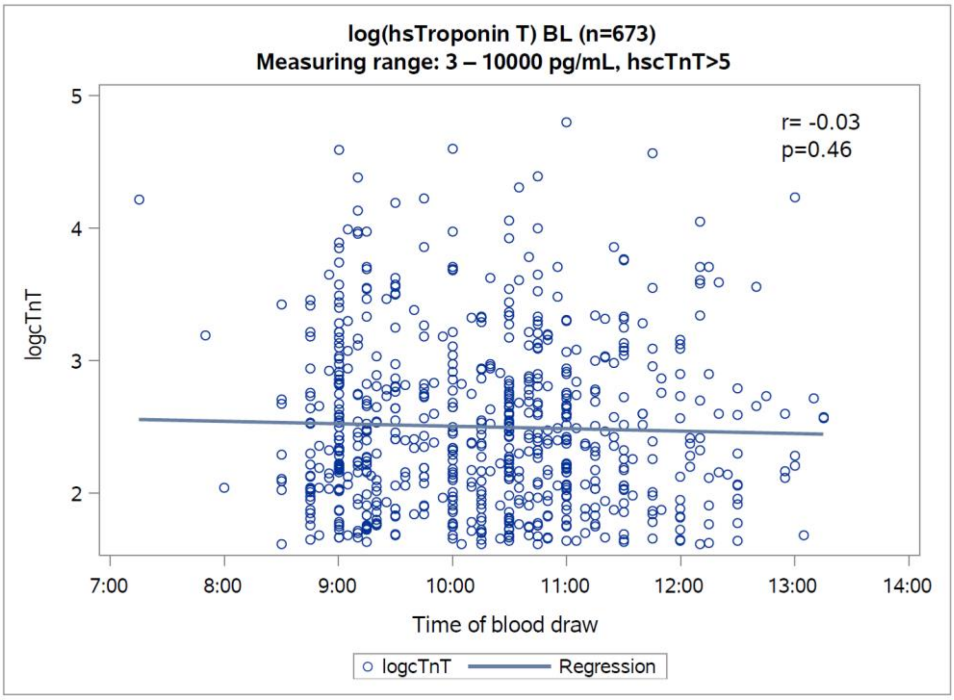

b)

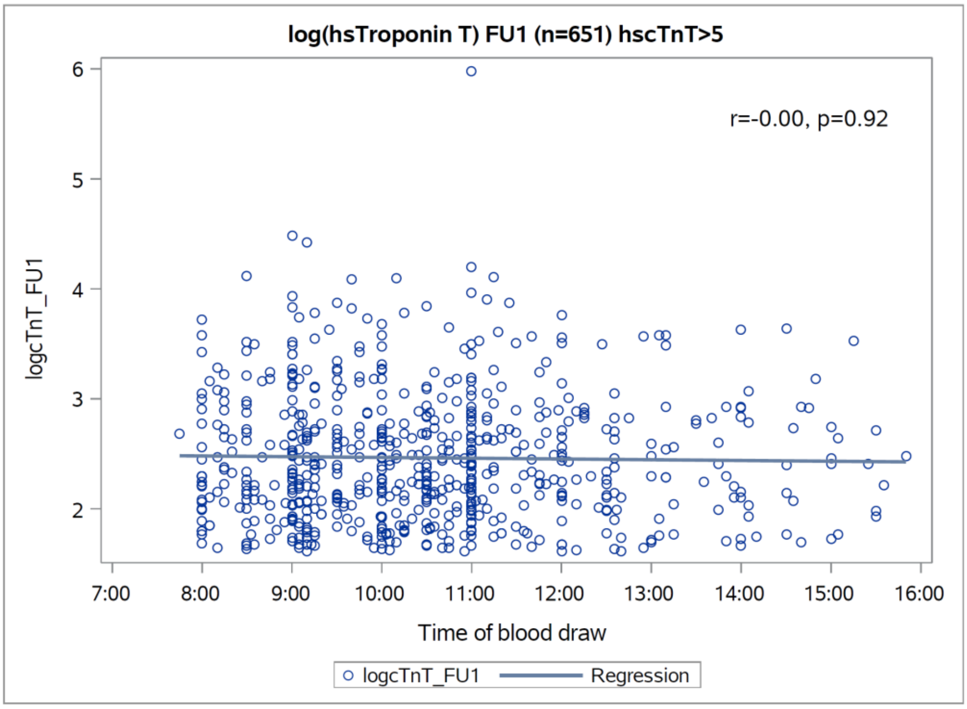

Supplementary Figure 2. Survival curves according to the 3-years Trajectories Groups for hs-cTnT using sex-specific cut-offs.

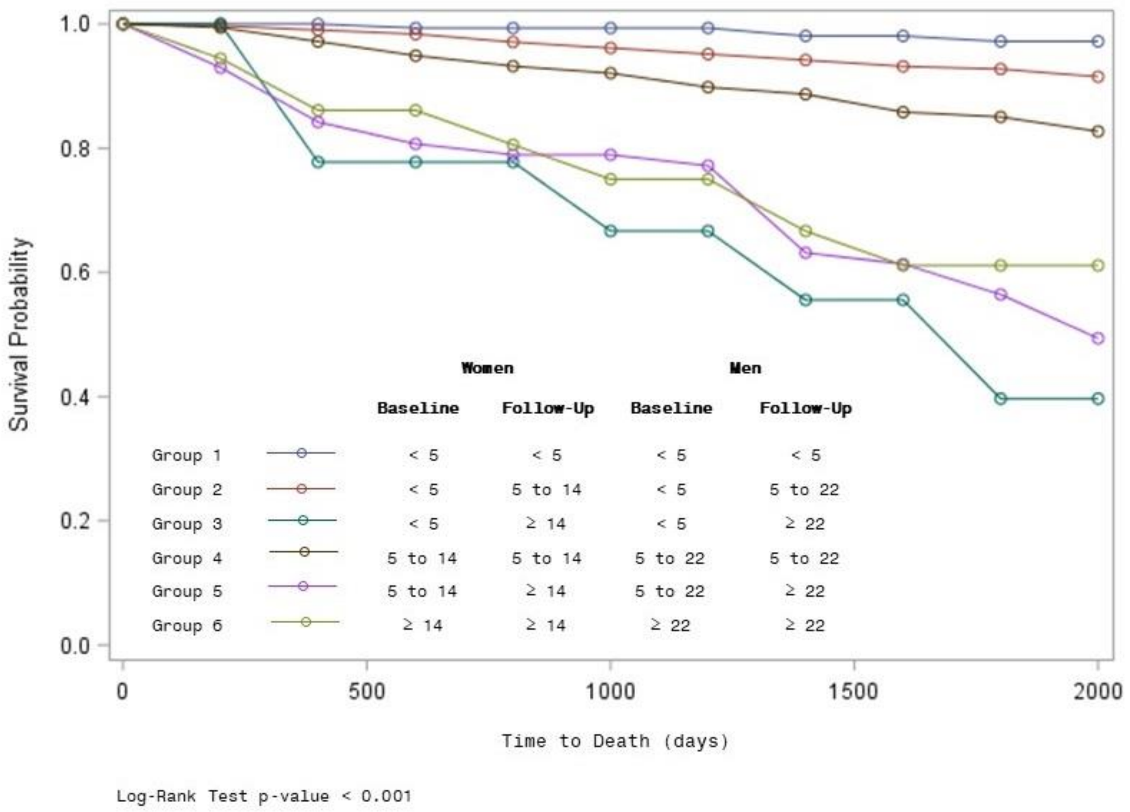

Supplement: Supplementary file 1 — Supplementary Material 1 [file 41598_2024_78641_MOESM1_ESM.pdf]
